# Surface Properties and In Vitro Corrosion Studies of Blasted and Thermally Treated Ti6Al4V Alloy for Bioimplant Applications

**DOI:** 10.3390/ma15217615

**Published:** 2022-10-29

**Authors:** Mohamed A. Hussein, Baha Y. Demir, Arumugam Madhan Kumar, Ahmed F. Abdelaal

**Affiliations:** 1 Interdisciplinary Research Center for Advanced Materials, King Fahd University of Petroleum & Minerals (KFUPM), Dhahran 31261, Saudi Arabia; 2 Department of Mechanical Engineering, King Fahd University of Petroleum & Minerals (KFUPM), Dhahran 31261, Saudi Arabia

**Keywords:** Ti alloy, biomaterials, thermal oxidation, corrosion

## Abstract

The biomedical Ti6Al4V alloy was thermally treated under sandblasting and mirror finish surface preparation conditions. The surface morphology, structure, roughness, wettability, and energy were characterized. Microhardness and in vitro corrosion studies were carried out. X-ray diffraction results showed a formation of rutile TiO_2_ phase for thermally treated samples under different pretreated conditions. The thermally oxidized samples exhibited an increase in microhardness compared to the untreated mirror finish and sandblasted samples by 22 and 33%, respectively. The wettability study revealed enhanced hydrophilicity of blasted and thermally treated samples. The surface energy of the thermal treatment samples increased by 26 and 32.6% for mirror surface and blasted preconditions, respectively. The acquired in vitro corrosion results using potentiodynamic polarization measurement and electrochemical impedance spectroscopy confirmed the surface protective performance against corrosion in Hank’s medium. The enhanced surface characteristics and corrosion protection of treated Ti6Al4V alloy give it potential for bio-implant applications.

## 1. Introduction

Titanium (Ti) and its alloys are frequently used as bio-implant materials in orthopedic and dental applications due to their low density, biocompatibility, and corrosion resistance [1,2]. The ability of Ti and its alloys to produce a stable passive oxide film is a significant factor in their selection, which provides remarkable corrosion protection. Ion accumulation on tissues next to the bio-implant [3] indicates that the native passive oxide can be destroyed at extremely low shear pressures [4]. During the fracture of the passive layer, wear debris and metal ions might cause unfavorable tissue reactions.

Ti6Al4V(Ti64) has been used in bio-implant applications in orthopedic and dental applications [1]. Surface characteristics of the bio-implants such as surface microstructure, coarseness, surface wettability, and energy of implants influence corrosion protection [5], and implant longevity [6]. Surface treatment can be performed on biomedical implants to alter their surface wettability and surface energy [7], and improve osseointegration [8]. Numerous surface modification techniques have been investigated, including chemical treatment [9], anodic oxidation [10], sol-gel [11], physical vapor deposition [12,13], laser treatment [14,15], ion implantation [16], and thermal oxidation [17,18]. Thermal oxidation is one of the simplest and most cost-effective processes for producing a barrier layer on Ti alloy. Thermal oxidation, which is frequently used to fabricate in situ ceramic coatings mostly composed of rutile, can result in the formation of thick, very crystalline oxide films and the subsequent dissolving of oxygen below them. Thermal oxidation of Ti alloys was reported for improving the hardness and wear resistance in biomedical applications [19,20,21,22]. Different surface treatments affect the implant’s corrosion resistance. The physiological environment is densely packed with protein, amino acids, and chemical compounds. The implant’s corrosion leads to the release of metal ions, which may affect its biocompatibility [23,24]. The surface features of Ti alloys have a critical role in corrosion behavior [25].

The composition and properties of the passive film in Ti biomedical alloys can change over time by reacting with ions and molecules and recreating the surface oxide film. This stability, as well as the ability to regenerate the passive film, is required for reduced ion release and, consequently, biocompatibility. The corrosion properties of pure Ti [26], TiNbZr and TiAlNb alloys [27], and Ti64 alloy in HCL solution [28] were investigated. After thermal oxidation treatment, the corrosion resistance of Ti6Al4V alloy in 20% HCl solution was reported to be improved [28]. The current density of thermally oxidized Ti6Al7Nb, Ti13Nb13Zr, and Ti15Zr4Nb alloys was reduced [27]. TO-treated titanium’s protective surface layer has been shown to last nearly 13 times longer than plasma-nitrided titanium [26].

Long-term interfacial contact between an implant and bone can be improved by coating the implant with a rough or porous surface to increase the available surface area for bone/implant apposition [29]. There are numerous methods for creating a rough surface on an implant surface [30]. Blasting is an effective method for increasing the surface roughness of metallic biomaterials and encouraging osseointegration. Blasting Ti6Al4V alloy has been identified as a low-cost method of increasing surface roughness [5,31,32,33]. Heat oxidation of commercially pure Ti implants appears to increase the percentage of bone-implant contact by the oxidized surface in vivo [34]. Thermal oxidation of polished Ti6Al4V improves initial cell attachment and cytoskeleton remodeling while only marginally increasing many osteoblast proliferation and differentiation markers [35]. The blasted and oxidized surfaces improved human osteoblast response [36] and pitting corrosion susceptibility [37].

Although there has been earlier research on the corrosion characteristics of untreated Ti64 alloy [38,39], the influence of surface finishing [40], and surface treatment [41] on Ti64 alloy in physiological solution [40], surface roughness on corrosion properties in HCL [42] limited studies were reported to examine the impact of surface pretreatment and thermal oxidation and on the surface properties and biocorrosion resistance of Ti64 alloy in physiological mediums. Thus, this research aims to study the surface pretreatment; sandblasting, and mirror-like and thermal oxidation of biomedical Ti64 alloy. The surface characteristics of the pretreated and thermally treated alloys were carried out; structure, morphology, surface wettability, and surface energy. The microhardness was evaluated using Vickers microhardness (HV). The biocorrosion analysis of the pretreated and oxidized samples was evaluated in Hank’s solution. Table 1 shows the manuscript’s symbols and abbreviations.

## 2. Experimental Procedure

### 2.1. Material, Surface Pretreatments, and Thermal Oxidation

Commercial Ti64 alloy was obtained from Xian Saite Materials Development Co., Xi’an, China, in a 32 · 25 · 3 mm^3^ size (chemical composition is depicted in Table S1 as per the standard). The surface-like mirror (M) was prepared by ground with 200, 400, 600, and 800 SiC followed by polishing with a suspension of 0.5 µm Al_2_O_3_ powder. Sandblasting (SB) was performed for 3 min at 0.55 MPa and 0.45 m^3^/min. The constant distance between the nozzle and the samples remained 7 cm throughout, and the nozzle was angled at 75°. Thermal oxidation was performed for both surfaces (M) and (SB) at a tube furnace (GSL-1700X, MTI, Tianjin, China). The samples were thermally heated at a rate of 10 °C/min to 700 °C for 4 h before being cooled to room temperature. The mirror-treated and blasted-treated samples were given the names MT and SBT, respectively. After the thermal treatment, the weight and area of the samples were measured to calculate weight gains per unit area. Figure S1 shows photograph for the Ti64 alloy treated at different conditions.

### 2.2. Structure and Surface Morphology Characterization

XRD was used to do a phase analysis of the samples (Rigaku, Kuraray, Japan) at a 30 kV and 15 mA. The 2θ angle was changed between 20 and 90° using a 0.02 step size and a 1.2°/min scanning rate. JEOL SEM (JEOL, Tokyo, Japan) was utilized to investigate the surface morphology. The surface elemental analysis was carried out using EDX (Oxford Instruments, Oxfordshire, UK).

### 2.3. Surface Analysis of the Treated Samples

An optical profilometer from Bruker Co (GTK-A, Bruker, Bellerica, MA, USA). was utilized to quantify surface roughness and surface topography. The measurement noise was removed from the raw measured surface topology data using a regular Gaussian regression filter [43,44]. For each measurement, at least five readings were taken in various regions. Wettability and surface energy were evaluated. To investigate wetting behavior, the optical contact angle measuring goniometer was utilized to determine the contact angle. The mean value of at least five contact angle measurements with water and glycerol (3 µL) was recorded for each measurement. Owens–Wendt was used to compute the surface energy [45,46] and as explained elsewhere [14,47]. To evaluate the reliability of the measurement results, the quality of the measurement results should be given as a quantitative indication. In this work, standard deviation was used to express the uncertainty of the measured surface roughness in terms of Ra and the optical contact angle. Type ‘A’ uncertainty evaluation standard was applied for Ra and optical contact angle uncertainty measurement.

### 2.4. Microhardness Measurement

Microhardness was determined utilizing the HV (Zwick Roell ZHV30, ZwickRoell, Romania Europe) hardness tester with a 500-gf load and a 15-s dwell time. Each sample was measured in a straight line at least seven times, and the average value was recorded.

### 2.5. Biocorrosion Study in Physiological Medium

Evaluation of in vitro corrosion-resistant of pretreated and thermally treated Ti64 samples was carried out in Hank’s solution using the Gamry Reference 3000 (Gamry Instruments, Philadelphia, PA, USA) potentiostat. Ti64 specimens with a contact area of 1.76 cm^2^ function as the working electrode, while the graphite rod and saturated calomel electrode (SCE) serve as the auxiliary and reference electrodes, respectively. The concentration of each component of Hank’s solution is given in Table 2. Before all the tests, stable OCP of the Ti64 samples was attained within 30 min in the tested medium. EIS tests were done using the frequency range from 100 kHz to 1mHz by applying 10 mV amplitude. Using a scanning rate of 1 mV/s, the potential range of −250 mV vs. OCP to 2000 mV vs. SCE was selected for PDP measurements. All obtained data were replicated three times to ensure their repeatability.

## 3. Result and Discussion

### 3.1. Analysis of The Weight Gain

Thermal treatment in the air (oxidation) begins with the absorption of oxygen molecules from the surrounding air and continues through oxide nucleation, the formation of a thin oxide film, and its development on a wider scale. Consequently, the sample’s mass will grow throughout the heat oxidation process. Normalized weight gain was determined from (dW/A) where dW is oxidized samples’ weight gain values, and A is the entire surface area. The weight increases for samples oxidized at 700 °C are 0.33 mg/cm^2^ for MT and 0.44 mg/cm^2^ for SBT, which is greater than the reported earlier value of 0.04 mg/cm^2^ [48]. This suggests that the SB sample surface absorbed more oxygen in this investigation when exposed to an air-oxidizing environment. The SB increases the surface area, and hence more area gets oxidized, and higher weight gain was observed.

### 3.2. Analyses of Structure and Surface Morphology

**Figure 1:**
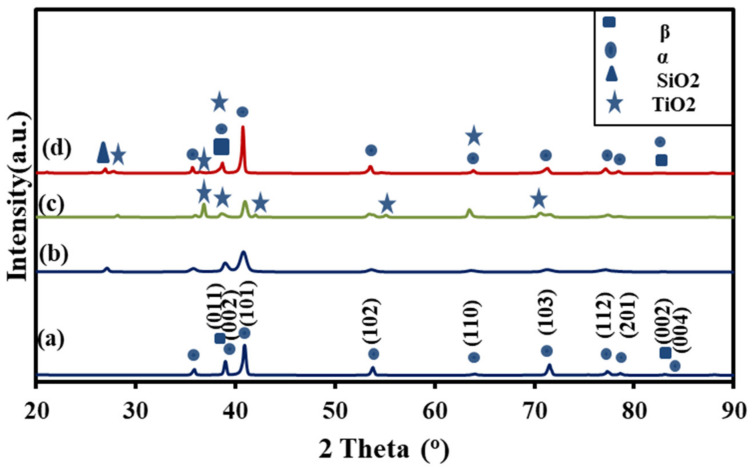
XRD of Ti64 alloy pretreated and thermally treated at various conditions (a) M, (b) SB, (c) MT, (d) SBT.

The phases of pretreatment and thermally treated samples were identified using XRD patterns. The M sample contained two phases (α + β) (Figure 1a), which is a typical structure for Ti64 alloys. The XRD after sandblasting contained (α + β) and SiO_2_, Figure 1b. The XRD of the SB sample revealed a broadening of the peaks in comparison to the M sample, reflecting a reduction in crystallite size, corroborating earlier findings [5,49]. The XRD patterns of the MT and SBT Ti64 alloy reveal the existence of TiO_2_ (rutile) along with α -Ti and β -Ti peaks. The results indicated that the oxide scale is composed of rutile TiO_2_. The morphology, chemical composition, and stable structure of TiO_2_ oxide scales, as well will affect the electrochemical reaction of the blasted, thermally treated samples in physiological mediums [18] and also enhance early bone formation [49,50].

**Figure 2:**
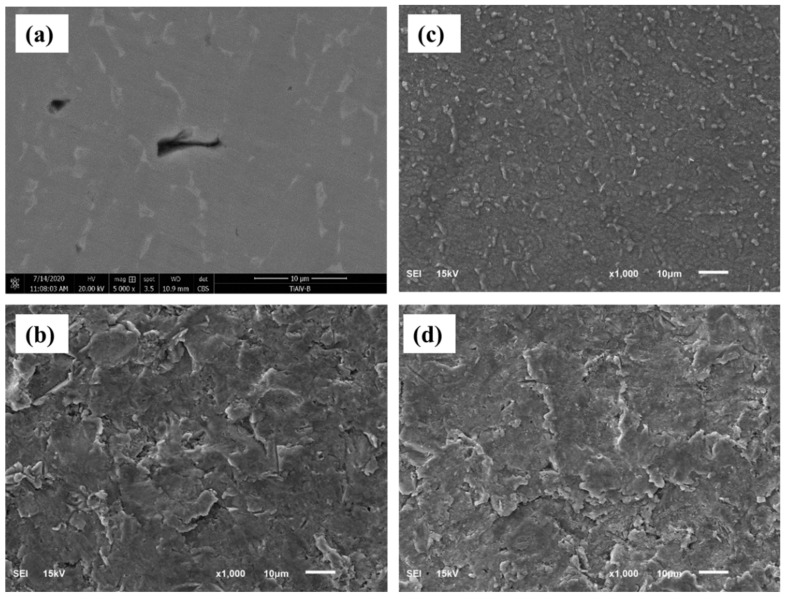
SEM micrograph of pretreated and treated Ti64 specimens (**a**) M, (**b**) SB, (**c**) MT, (**d**) SBT.

**Figure 3:**
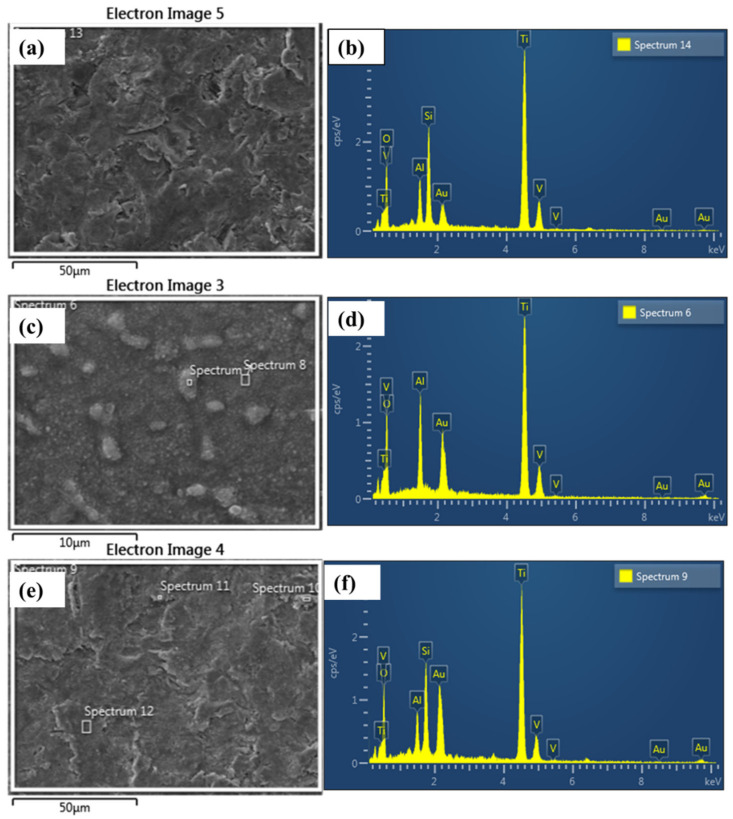
EDX results of Ti64 treated at different conditions; (**a**,**b**) SB; (**c**,**d**) MT; (**e**,**f**) SBT.

Figure 2 illustrates the morphology of the pretreated and thermally treated Ti64 surface. Untreated alloy surface (Figure 2a) showed the existence of two phases (α + β) for Ti64 alloys with a smooth flat surface as the surface was not etched. Figure 2b demonstrates a significant change in the microstructure of SB, as the smooth, flat surface was replaced with coarse, rough morphologies resembling pits and valleys. This is often created as a result of silica particles impinging on one another. SB was found to include Si and O as a result of silica particle remains on the surface employed in the sandblasting method as validated from the XRD examination (Figure 1) and EDX results (Figure 3). The SEM micrographs of thermally oxidized specimens demonstrate the absence of spallation and the presence of oxide scales (Figure 2c,d). As may be seen, the untreated surface is devoid of oxide scales. On the other hand, the substrate is covered with multiple thin and microscopic oxide scales and islands, and huge grains of TiO_2_ emerge from oxide aggregation for MT, thickening the scale. Moreover, the oxide scales are dense and evenly distributed across the surface, indicating a homogeneous coating. The production of an oxide film begins with the development of a thin oxide scale, followed by its aggregation and total surface coverage. Spallation of the oxide scales has been observed in the literature for oxidization at 800 °C [18]. The history of the surface morphology of the oxide layer formed on Ti64 thermally oxidized samples demonstrates that when oxide comes into direct contact with oxygen, it nucleates throughout the surface of the specimens.

### 3.3. Surface Roughness and Topography

**Figure 4:**
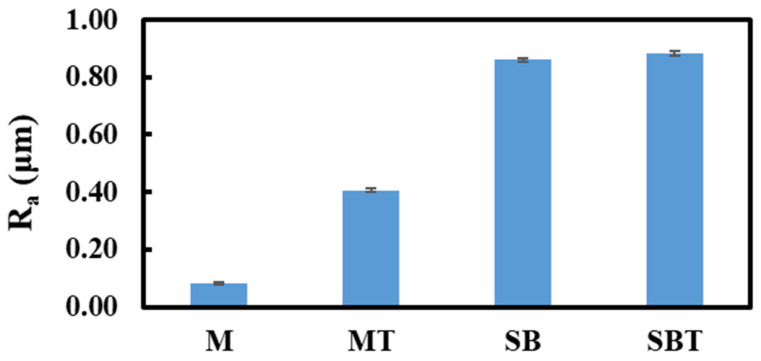
The roughness of pretreated and treated Ti64 samples at different conditions.

**Figure 5:**
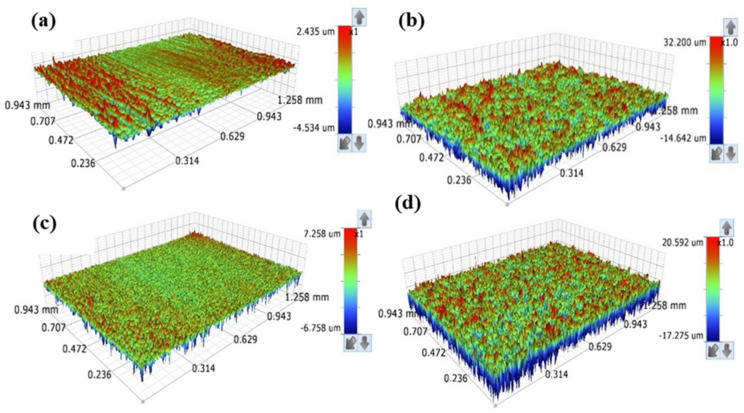
3D surface topography for pretreated and treated Ti64 alloy: (**a**) M, (**b**) SB, (**c**) MT, (**d**) SBT.

Figure 4 depicts the optical profilometer results of pretreated and thermally oxidized Ti64 samples. The surface of the M sample was smooth, but the SB sample was rough. The arithmetic means height was employed to quantify the surface’s roughness (Ra). The measured values of Ra have a maximum uncertainty value of 0.018 µm. SB increased the surface roughness of the untreated sample to 0.861 µm. Due to the interaction of sand particles with the surface, the sandblasting process produced a surface with a relatively high degree of roughness. As indicated in Figure 5b, the alloy surface was plastically deformed during the sandblasting technique. As a result, SB samples exhibited a considerable change in the topography, characterized by uneven features and coarseness. Although the profile of the base alloy is softer and more uniform, the profile of the sandblasted surface was jagged, with valleys that emerged during the sandblasting process causing considerable deflections. The MT sample showed a Ra value of 0.407 µm which is five times that of the M. However, the SBT did not show a significant variation in the roughness related to the SB sample. Following thermal oxidation, the increase in surface roughness of the Ti64 alloy is related to the development mechanism of the oxide film. Temperature increases the kinetics of outward expansion, resulting in higher roughness values. The high surface roughness of thermally treated samples is a result of strong internal stress in the oxide film, mismatching lattices, and a significant difference in thermal expansion coefficients between the rutile phase and titanium [17]. It is hypothesized that rough-textured biomaterial surfaces promote osteoblast cell adhesion and development [51,52]. Increased surface roughness increases the number of grain boundaries per unit area and enhances cell adhesion [53]. Roughness and alloy surface properties were reported to be essential for cell activity [54], and cell adhesion was found to be dependent on titanium oxide thickness, micro porosity, and surface roughness [37].

### 3.4. Microhardness

**Figure 6:**
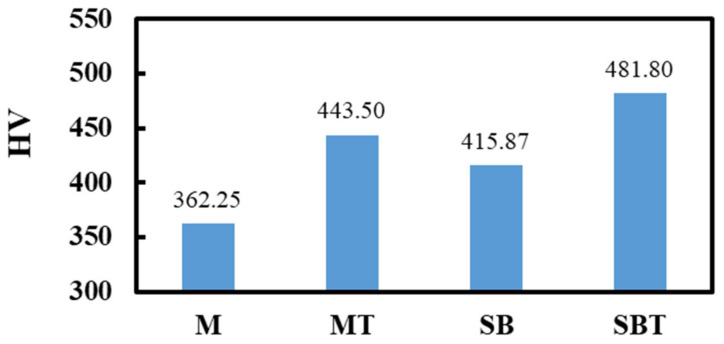
Microhardness of pretreated and treated Ti64 alloy at different conditions.

The microhardness measurements of the thermally treated and pretreated samples are shown in Figure 6. Untreated mirror Ti64 has a microhardness of 362 HV. The results demonstrated that oxidation treatment results in a rise in microhardness. The MT specimen heated to 700 °C exhibits a 22% increase in hardness over the M sample, reaching 443.2 HV. The increase in hardness is due to the formation of a hard oxide coating and the strain. Compared to the M specimen, SB increases the hardness by around 15% due to plastic deformation and fine crystal formation. The SBT shows a higher increase in HV by 33% compared to the M sample. The microhardness of thermally oxidized Ti64 alloy increases as a result of the formation of hard oxide layers and stresses generated by oxygen’s solubility beneath the oxide layer of the substrate [55,56].

### 3.5. Wetting Behavior and Surface Energy Evaluation

**Figure 7:**
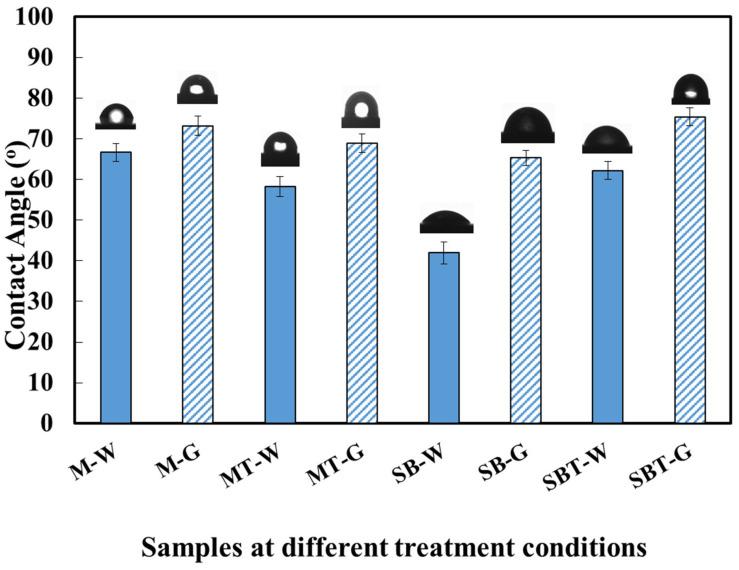
CA measurement for pretreated and thermally oxidized Ti64 alloy; water (W), glycerol (G).

To have a better understanding of the interaction between the treated surface and physiological media, wettability studies were conducted. Wettability affects cell adhesion. Utilizing contact angle (CA) measurements, the hydrophilicity of the surface was estimated. Figure 7 illustrates a water droplet alongside the expected contact angle for untreated and treated Ti64 alloy samples of DI water and glycerol. CA values have a maximum uncertainty of 2.63°. CA values for SB, MT, and SBT samples were lower than those for the M sample indicating enhanced wettability by blasting and thermal oxidation. The M sample had CA ~66.68° and 73.23° using water and glycerol, respectively. The SB sample had CA of ~41.93° and 65.29° using water and glycerol, respectively. The heat treatment process affected the CA measurement for M. Due to the development of the oxide phase during thermal oxidation, the CA dropped.

After three minutes of sandblasting, the water contact angle (WCA) was reduced as the surface roughness of the SB specimen increased, and the surface area increased. The trend of the data is consistent with a recent publication [57], which shows that when the implant surface area increases, the CA decreases. SB and MT improve the surface’s wettability in proportion to the increased surface area exposed as a result of increased surface roughness. The results indicated that the treated and thermally oxidized treatment decreased the water contact angle, indicating that a hydrophilic surface may be achieved through surface modification. SB had a greater FWHM than bare specimens (Figure 1c,d), indicating that the crystallites in SB were smaller than those in the untreated sample. Consistent with prior research [58], the wettability results demonstrated that the contact angle decreases as the crystallite size decreases. Surface energy has a substantial effect on the intermolecular interactions, surface wetting, and adsorption behavior of a material with other compounds. Using the Owens–Wend method, the surface energy of untreated and treated samples was determined by measuring the contact angles of water and glycerol. Table 3 lists the contact angle and surface energy. The surface energy of the samples ranges from 38.2 to 81 mJ/m^2^. Surface moisture influences cell adhesion and proliferation significantly [59]. The wettability of the surface influences its surface energy. According to the Wenzel formula [60], increasing the surface energy increases the interaction of liquids and proteins with titanium. The surface energy of the thermal treatment samples (MT, SBT) increased by 26% and 32.6% compared to the mirror surface and blasted preconditions, respectively. The hydrophilic surface characteristic was reported to boost cell interaction and biocompatibility [61,62].

### 3.6. In Vitro Corrosion Study in Hanks Solution

**Figure 8:**
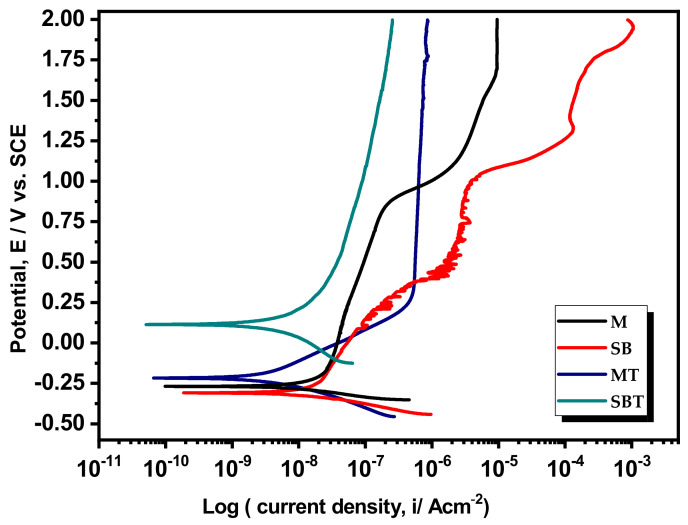
PDP plots of the pretreated and treated Ti64 specimens in Hank’s solution.

Characteristic PDP curves of treated Ti64 samples are depicted in Figure 8. The estimated electrochemical values, for example, the corrosion current dentistry (i_corr_), corrosion potential (E_corr_), and passive current density (i_p_) are given in Table 4. All the investigated substrates displayed nearly similar cathodic branches, revealing that the reduction reaction that occurred in the cathodic area is almost the same with different rates [12]. In contrast, notable differences with passive characteristics were detected in the anodic branches of the PDP curves. Specimen M (Figure 8) showed a slight anodic slope from −0.195 to 0.750 V, signifying the dissolution of Ti64, and then, it was almost perpendicular with a minor slope that became constant until 2 V. This observation is possibly linked to the lesser oxygen diffusion due to the passive film on the Ti64 specimen [63]. This treatment results in the production of an active surface with a rough surface, Specimen SB exhibited a nearly identical behavior, characterized by a substantial fluctuation in the anodic branch. Specimen SB exhibited a nearly identical behavior, characterized by a substantial fluctuation in the anodic branch. It has already been reported that the active surface with high surface roughness caused by the SB treatment normally exhibited some fluctuation in the anodic region of the PDP curve [5].

In contrast, the thermally treated specimens, SBT and MT showed different trends compared to those of the M and SB specimens. The anodic slope is observed to be vertical, representing the reduction in anodic dissolution. Moreover, unlike specimens M and SB, thermally treated specimens displayed a lower current density in the segment stretching from the onset of anodic polarization to about 1000 mV and maintaining constant up to 2000 mV. Therefore, the attained results indicated that the thermally treated specimens displayed a passive state in Hank’s solution, whereas specimens M and SB revealed an active behavior. From Table 4, it is clear that i_corr_ of the thermally treated specimens was observed to be reduced by 47.28% and 72.69% compared to that of specimens M and SB. Furthermore, the passive current density (i_p_) of the thermally treated specimens is also lower than that of the M and SB specimens (~60 μA cm^−2^). 

The observation of a larger passive region on the SBT specimen confirmed that the passivation layer of the sandblasted and thermally treated samples produced a compact, ordered, and structurally stable passive oxide layer on the surface of the Ti alloy, which exhibited superior corrosion-resistant performance compared to the M and SB specimens.

**Figure 9:**
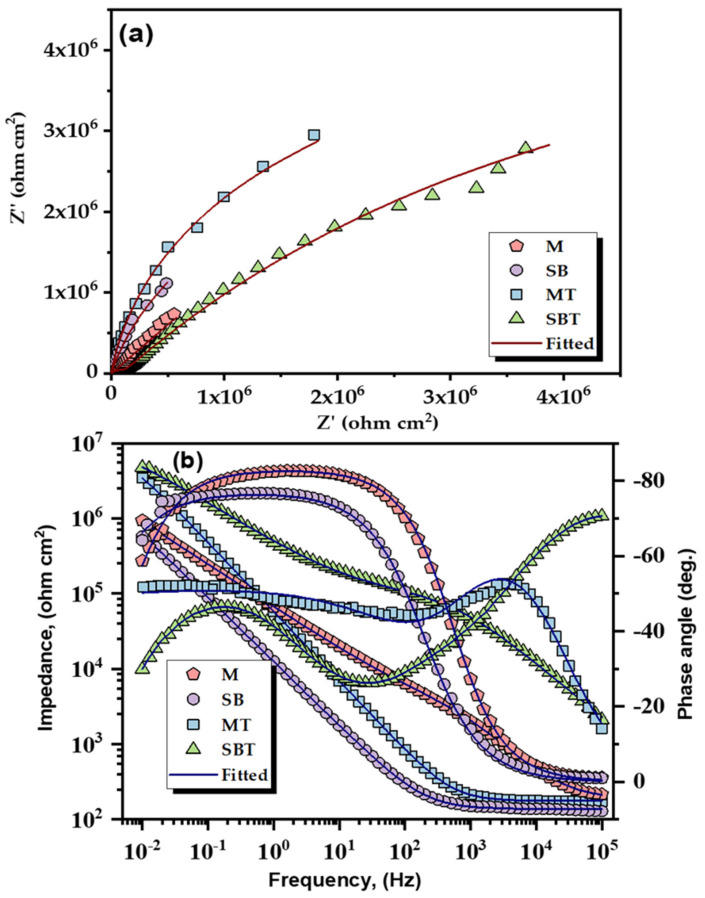
(**a**) Nyquist and (**b**) Bode plots of pretreated and treated Ti64 samples in Hank’s solution.

Figure 9a presents the Nyquist curves of treated Ti64 specimens in Hank’s medium, representing the evolution of measured impedance. The experimentally obtained and the simulated EIS curves are symbolized by solid signs and lines, respectively. Nyquist plots of treated specimens exhibited a distorted capacitive arc with linear-like behavior in the tested electrolyte, suggesting that the treated surface hinders the charge transfer phenomenon at the metal/electrolyte interface. It is observed that the various quarter-arcs depicted in the Nyquist graphs of Figure 9a deduce the capacitive properties, whose radii represent the corrosion resistance [64]. Figure 9b presents representative EIS charts for treated Ti64 samples in Bode resistance and phase angle formats. The Bode curves of all studied specimens exhibited two separate parts, the one associated with high frequencies illuminating constant impedance values and a lower phase angle denoting solution resistance. When the frequency shifts in the medium and low-frequency regions, the impedance value increases gradually with the increase in phase angle and exhibits a slope of around −1, confirming the capacitive response of studied Ti64 samples. The impedance modulus, |Z| at 0.01 Hz, reveals the polarization resistances of the specimens, which are greater for the thermally treated specimens, indicating superior corrosion resistance performance. In addition to quantitatively estimating the acquired EIS results, the EIS circuit fitting operation was carried out utilizing the Echecm analysis program and the appropriate EIS model.

By thoroughly inspecting the EIS curves with one-time constant behavior, modified Randle’s circuit [R_s_ (R_ct_CPE_dl_)] was engaged in fitting the experimental EIS curves. The employed EIS model was comprised of the solution resistance (R_s_), and charge transfer resistance (R_ct_) along the constant phase element (CPE) of double-layer capacitance. CPE has replaced the capacitor, compensating for the non-ideal behavior of examined specimens due to the impact of surface heterogeneities of the specimens [65,66]. The chi-squared (χ^2^) values associated with the goodness of fit were observed to be in the range of 10^−4^, illuminating the satisfactory fineness of fitting for the nominated EC model. The extracted parameters from the circuit fitting are summarized in Table 4. The value of R_ct_ is generally the resistance of the charge transfer through the oxide film and directly controlled through the anodic dissolution and higher R_ct_ often represents the enhanced corrosion resistance of the metallic material. From Table 4, the thermally treated Ti64 specimens in Hanks showed higher values of R_ct_, which are increased by about one order of magnitude compared to that of specimens M and SB, revealing the enhanced barrier characteristics provided by the passivated film on Ti64 specimens. Moreover, the thermally treated specimens displayed a remarkable reduction in CPE_dl_ values by one order of magnitude, corroborating the significant reduction in the permeation of hostile ions from the Hanks solution. Hence, the effective advantage of sandblasting followed by thermal treatment on Ti specimens, related to that of untreated Ti64 samples in terms of electrotechnical corrosion performance, is designated by the acquired higher R_ct_ with lower CPE_dl_ values and reduced i_corr_ with lower i_p_ values, therefore confirming the efficacy and electrochemical stability of the treated Ti64 surface in Hank’s solution. From the acquired EIS and PDP results, the corrosion resistance of the examined Ti64 specimens can be categorized as follows, SB < M < MT < SBT.

## 4. Conclusions

The thermal treatment of Ti64 alloy at 700 °C for 4 h for two different surfaces under pretreated conditions, sandblasted and mirror finish, was studied. The effect of surface pretreatment and thermal treatment on surface energy, microhardness, and in vitro corrosion characteristics was studied. The in vitro electrochemical corrosion analysis was carried out in Hank’s medium. The following conclusions have been obtained:

1. Structure analysis reveals a formation of rutile TiO_2_ phase for thermally treated samples at M and SB pretreated conditions.
2. The thermally oxidized samples showed improvements in microhardness by 22% and 33% for the M and SB samples, respectively.
3. The hydrophilicity of Ti64 alloy was enhanced by blasting and thermal treatment and the surface energy of the thermal treatment samples increased by 26% and 32.6 for mirror surface and blasted preconditions, respectively.
4. Acquired in vitro electrochemical corrosion result confirmed the favorable role of thermal treatment and sandblasting (SBT) of Ti64 alloy by exhibiting higher impedance (4820.89 kΩ cm^2^) with lower capacitance (0.075 Ω^−1^ cm^−2^ s^n^) values in EIS and reduced i_corr_ (0.0060 µA cm^2^) with lower i_p_ (0.1102 µA cm^2^) values in PDP results in comparison with the M and SB specimens.
5. The results indicate that the thermally treated Ti64 biomedical alloy has enhanced surface properties and in vitro electrochemical corrosion resistance and may have potential uses in biomedical applications.
6. Future research is proposed to include a bioactivity study, an in vitro corrosion study with a long immersion time, and an XPS surface analysis.

## Figures and Tables

**Table 1:** Symbols and abbreviations used in the manuscript.

Symbol/Abbreviations	Description
Ti64	Ti6Al4V
M	the surface-like mirror
SB	sandblasting
MT	thermally treated surface-like mirror
SBT	thermally treated sandblasting
XRD	X-ray diffraction
SEM	scanning electron microscope
EDX	energy dispersive X-ray
HV	Vickers hardness
SCE	saturated calomel electrode
OCP	open circuit potential
EIS	electrochemical impedance spectroscopy
PDP	potentiodynamic polarization measurement
(α + β)	(Alpha + beta)
Ra	surface’s roughness
CA	contact angle
W	water
G	glycerol
FWHM	full width at half maximum
$\gamma_{s}^{p}$	polar component for the surface energy
$\gamma_{s}^{d}$	dispersive component for the surface energy
$\gamma_{s}$	total surface energy
Ecorr	corrosion potential
ip	passive current density
icorr	corrosion current dentistry
Rs	solution resistance
Rct	charge transfer resistance
CPE	constant phase element

**Table 2:** Composition of Hank’s solution.

Components	Concentration (g/L)
CaCl_2_ (anhydrous)	0.14
MgSO_4_ (anhydrous)	0.10
KCl	0.40
KH_2_PO_4_	0.06
NaCl	8.00
Na_2_HPO_4_ (anhydrous)	0.048
NaHCO_3_	0.35
D-Glucose (Dextrose)	1.0

**Table 3:** Surface energy for pretreated and thermally treated Ti64 specimens.

Sample	$\gamma_{s}^{p}$ $\left(mJ/m^{2}\right)$	$\gamma_{s}^{d}$ $\left(mJ/m^{2}\right)$	$\gamma_{s}$ $\left(mJ/m^{2}\right)$
M	35.6	2.6	38.2
SB	81	0.07	81.0
MT	46.5	1.73	48.2
SBT	50.4	0.26	50.6

**Table 4:** Electrochemical parameters extracted from the corrosion tests in Hank’s medium.

Specimen	E_corr_ V	I_corr_ A cm^2^ × 10^−7^	i_p_ A cm^2^ × 10^−7^	R_s_ Ω cm^2^	R_ct_ kΩ cm^2^	CPE_dl_	
						Y0 Ω^−1^ cm^−2^sn × 10^−6^	n_dl_
M	−0.244	0.173	96.354	125.56	952.25	1.894	0.99
SB	−0.293	0.223	119.525	142.52	654.86	15.548	0.94
MT	−0.201	0.0912	6.705	135.26	3491.25	0.154	0.98
SBT	−0.112	0.0609	1.102	141.86	4820.89	0.075	0.98

## Data Availability

All relevant data were used in this manuscript.

## Supplementary Materials

The following supporting information can be downloaded at: https://www.mdpi.com/article/10.3390/ma15217615/s1, Figure S1: Photograph of the pretreated and thermally treated samples (a) M, (b) SB, (c) MT, (d) SBT; Table S1 the chemical composition of Ti6Al4V–Grade 5–ASTM F136.
