# Supplementary material for: Surface Properties and In Vitro Corrosion Studies of Blasted and Thermally Treated Ti6Al4V Alloy for Bioimplant Applications
Source: Materials (Basel). 2022 Oct 29;15(21):7615. doi: 10.3390/ma15217615 (PMC9655274; doi:10.3390/ma15217615)
Supplement: Supplementary file 1 [file materials-15-07615-s001.zip › materials-1977335-supplementary.pdf]

materials MDPI

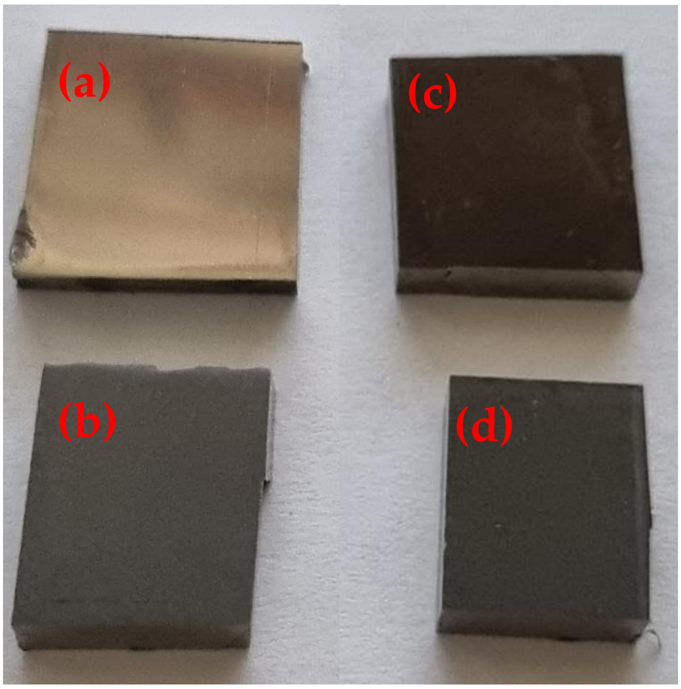

Figure S1. Photograph of the pretreated and thermally treated samples (**a**) M; (**b**) SB; (**c**) MT; (**d**) SBT.

Table S1. the chemical composition of Ti6Al4V- Grade 5 – ASTM F136.

**Material**	**Ti**	**Al**	**V**	**Fe**	**O**	**H**	**C**	**N**
Composition, Wt. %	Bal.	5.5–6.50	3.5–4.5	≤ 0.25	≤ 0.13	≤ 0.012	≤ 0.08	≤ 0.05
